# Editorial: Recent Advances in Psychiatry From Psycho-Neuro-Immunology Research: Autoimmune Encephalitis, Autoimmune Encephalopathy, and Mild Encephalitis

**DOI:** 10.3389/fpsyt.2019.00169

**Published:** 2019-03-29

**Authors:** Karl Bechter, David Brown, Souhel Najjar

**Affiliations:** ^1^Department of Psychiatry and Psychotherapy II, Bezirkskrankenhaus Guenzburg, University of Ulm, Ulm, Germany; ^2^Department of Immunopathology, ICPMR, Westmead Hospital, Westmead, NSW, Australia; ^3^Department of Neurology, Zucker School of Medicine at Hofstra/Northwell, Lenox Hill Hospital, New York, NY, United States

**Keywords:** neuroinflammation, mild encephalitis, autoimmune encephalitis, encephalopathy, schizophrenia, affective disorders, depression, autoimmune psychosis

The precise etiopathophysiology of most severe mental disorders, particularly schizophrenia spectrum and affective spectrum disorders, remains unclear but is thought to be a product of the intricate interplay among a number of risk factors. These include interactions between genetic, developmental and environmental factors, such as infections. Indeed, the evidence from many epidemiological studies carried out in different countries, especially those from Denmark, strongly associates both early and the frequency and severity of life-time infections with an increased risk of psychosis in later life, possibly through diverse immunological mechanisms. More recently, the continued discovery of antibodies against various neuronal cell surface proteins, such as anti-N-methyl-d-aspartate receptor (NMDAR) and gamma-aminobutyric acid beta receptor (GABAßR), directly links central nervous system (CNS) autoimmunity with dysregulation of glutamatergic and GABAergic neurotransmitter pathways to the neurobiology of acute psychosis in individuals with autoimmune encephalitis (AE). These findings, along with the rapidly emerging evidence of many other immunological abnormalities in most severe mental disorders, have made it clear that the nexus of psychiatry, neurology, and neuroimmunology represent a fruitful coalescence in understanding the pathogenesis of psychiatric disease. The interactive presentations on the link between immune dysregulation and various mental disorders during the 13 and 14th Psychoimmunology expert meetings in 2016 and 2018 at Ulm University in Germany (www.psychoimmunology-experts.de) generated widespread interest and discussion among the participants, which led to identifying and planning this research topic. It is comprised of 23 peer-reviewed articles, including original research, reviews, opinion pieces, and case reports.

Here, we provide a brief summary of the main findings of the papers included in this research topic that link inflammatory and immunological mechanisms to the neurobiology of psychiatric symptomatology.

Herken and Pruess reviewed the neurological and psychiatric presentations of 100 patients recruited from the Charité Centre for autoimmune encephalitis in Berlin. In this cohort, about 60% of the individuals presented mainly with psychiatric symptoms that remained dominant throughout the clinical course. About one-third of those patients were initially hospitalized for psychiatric evaluation and treatment. All patients with anti-NMDR encephalitis exhibited behavioral disturbances, hallucinations, delusion, short-term memory deficits, or catatonia. Individuals with other neuronal antibodies were also frequently admitted with psychosomatic diagnosis. The authors identified so-called “red and yellow flags” to facilitate early recognition of patients with autoimmune encephalitis presenting with neuropsychiatric disturbances, highlighting the usefulness of incorporating cerebrospinal fluid (CSF) analysis in the standard diagnostic workup.

Al-Diwani et al. Oxford UK, proposed using a syndrome-level taxonomy for isolated psychiatric syndromes associated with neuronal antibodies that can overlap with autoimmune encephalitis presenting with neuropsychiatric presentations; synaptic and neuronal autoantibody-associated psychiatric syndromes or “SNAps.” This pragmatic approach can serve as a reminder to consider early autoantibody screening for diagnosing SNAps, potentially permitting early diagnosis and management of these rare forms of autoimmune encephalitis that can mimic intractable severe mental disorders. Additional research is needed to investigate the therapeutic implications of SNAps and validate their presence as distinct clinical entities.

Ellul et al. provided a thorough review of the clinical evidence supporting the existence of autoimmune psychosis as a distinct clinical entity among individuals with new-onset psychosis, highlighting the diagnostic challenges and therapeutic implications associated with this entity. The authors reviewed the clinical and biological features of autoimmune psychosis, including peripheral biomarkers of autoimmune dysfunction from dysbiosis to autoantibodies such as NMDAR antibodies, discussing the interplay between environmental and genetic factors.

Najjar et al. summarized the clinical and experimental findings suggestive of the potential contribution of neurovascular unit dysfunction and blood brain barrier hyperpermeability to the neurobiology of schizophrenia. These include neuroinflammation- and oxidative stress-related neurovascular changes including endothelial dysfunction, leading to increased cross interactions between brain innate and peripheral adaptic immunities, thereby perpetuating harmful inflammatory and immune responses in the CNS. The authors concluded that these findings provide additional support for the mild encephalitis (ME) hypothesis of schizophrenia.

Borroto-Escuela et al. reviewed the molecular data supporting the volume transmission hypothesis with specific relevance to NMDAR and its pathological allosteric receptor-receptor interactions that can lead to increased internalization and decreased NMDAR signaling. Combined with the triplet puzzle theory it is suggested that mild neuroinflammation is associated with formation of D2R heteromers, which in turn can enhance D2R promoter signaling, leading to schizophrenia-like symptoms.

de Haan et al. reviewed the chronic self-sustaining immunological and inflammatory changes associated with neurodegenerative and psychiatric disorders. The findings link the cascading effects of neuroinflammation and autoimmunity to disturbances of cholinergic, dopaminergic, glutamatergic, histaminic, and serotonergic functions, relevant to the pathophysiology of neuropsychiatric disturbances associated with neurodegenerative and psychiatric disorders. Thus, these related neuropsychiatric disorders might benefit from novel immunotherapies.

De Picker et al. presented a meta-review of recent quantitative systematic reviews and meta-analyses from 2010 to 2017 investigating the functional relevance of microglial activation-related immune signaling and brain plasticity to the pathophysiology of acute psychosis and schizophrenia. This review included data derived from translocator protein (TSPO) positron emission tomography, CSF analysis, and post-mortem studies, coupled with the results of clinical trials pertaining to the efficacy of various immuno-modulatory agents. They suggested that microglial activation and its downstream effects on the immune processes and neuroplasticity can influence the clinical presentation and the course of schizophrenia. However, the functional relevance of the cross talks between systemic and brain inflammation is less clear.

Riedmüller and Müller and Müller and Riedmüller discussed the potential ethical implications of the ME hypothesis of schizophrenia that includes shifting our perspective on schizophrenia from being an incurable psychiatric syndrome to a chronic but a treatable neurological disease. This will lead to a newer theoretical conceptualization of schizophrenia that necessitates interdisciplinary care teams to diagnose and manage new-onset psychosis and schizophrenia. Moreover, this reform will have potential repercussions for the pharmaceutical industry and legal implications surrounding current compulsory treatment orders. It might also limit social isolation and decreases the burden and stigma associated with severe mental disorders.

Kočovská et al. summarized the role of vitamin D deficiency as a potential environmental risk factor for three etiologically distinct disorders; multiple sclerosis, schizophrenia, and autism. The data suggest that vitamin D deficiency has a much more robust role in MS, compared with that in schizophrenia and autism. Further, Endres et al. investigated the prevalence of vitamin D deficiency in a 1-year cohort of adult inpatients with schizophreniform (*n* = 60) and autism spectrum syndromes (*n* = 23) at German tertiary care hospital, compared with that in control group (*n* = 3,917). Severe deficiencies (< 10 ng/ml) were found more frequently in the schizophreniform (38.3%) and autism spectrum groups (52.2%), compared to that of control group (16.3%). These findings justify the need for a more frequent assessment of serum vitamin D levels in these disorders, and advocate for additional randomized clinical trials investigating the effectiveness of vitamin D supplementation in ameliorating psychiatric and behavioral symptoms.

Endres et al. investigated the prevalence of CSF inflammatory abnormalities in a seven-year cohort study at the Freiburg University clinic. In 63 patients with bipolar disorder, CSF abnormalities, suggestive of mild inflammation, were found in 19% of patients. These include increased albumin quotients (12.9%), increased immunoglobulin (Ig) G indices (3.2%), oligoclonal bands (OCBs) in 1.6%, increased white blood cell count (1.6%). These findings further support the ME hypothesis of schizophrenia and autoimmune encephalopathy masquerading as severe mental disorder.

Vogels et al. verified the previously reported findings of T-cell deficits and monocyte immune gene activation in a well-controlled cohort of 97 individuals with largely euthymic bipolar disorder. Notably, within the decreased T-cell populations, the counts of T-helper 17 and T-helper 2 were increased whereas T regulatory cell counts were decreased. Moreover, the circulating monocytes demonstrated an increased frequency of anti-inflammatory phenotypes. The authors conclude that T-cell deficits are likely a trait phenomenon while pro and anti-inflammatory factors are state dependent in individuals with bipolar disorder.

Krause et al. showed significantly lower serum kynurenine level, and higher quinolinic acid/kynurenine (Qui/Kyn) ratio in a cohort of 32 individuals with major depression, compared to 20 healthy controls. Higher baseline kynurenine/tryptophan (KYN/TRP) ratio at baseline was predictive of remission and lower Qui/Kyn ratio following an add-on treatment with celecoxib correlated positively with remission. This study suggests that the measurements of above biomarkers might be useful in selecting individuals with major depression who are more likely to respond to anti-inflammatory agents.

Ajdacic-Gross et al. analyzed epidemiological data from the PsyCoLaus, a large cohort study (*n* = 3,720) in Switzerland, to explore the potential association between various early-onset anxiety disorders and the age at the onset of common viral childhood illnesses such as chickenpox, measles, and mumps. The authors found that only social phobia among early anxiety disorders was associated with delayed-onset viral infections, suggesting that common viral childhood infections of a delayed onset may increase the risk of social phobia, speculatively by infection-induced behavioral changes.

Rahman et al. investigated the developmental effect of maternal immune activation (MIA), during early and late gestation, on glutamatergic signaling via NMDAR-related molecular changes in various brain regions (cortex, hippocampus, and striatum) in adult rat offspring. The authors found that MIA can alter NMDAR indices, such as increased NR2A expression in cortical and hippocampal regions. This was more prominent in male compared with female offspring, irrespective of MIA gestational timing. They concluded that MIA-induced developmental molecular changes including increased NR2A expression in male offspring might trigger enduring vulnerability to impaired neuroplasticity and its consequent behavioral changes.

Sommer et al. investigated the neural effects of the hydrogen sulfide (H_2_S, a gaseous molecule), which is endogenously produced by enzymes utilizing cysteine in the peripheral and central nervous systems. The authors also conducted another experiment to analyze the effects of various antipsychotics on the expression of H_2_S forming enzymes in human cell lines. Local sodiumhydrogensulfide (NaHS) infusion alone into the hippocampus resulted in a significant increase in the hippocampal glucose and lactate levels, as well as glutamate release. Pretreatment with peripheral inflammatory lipopolysaccharide (LPS) was associated with a further increase in lactate, but without altering glutamate levels. While NaHS infusion was associated with a significant increase in hippocampal free radical formation, by contrast, LPS pretreatment was associated with reduced radical formation. Additionally, neuroleptics exhibited differential effects on the H_2_S forming enzymes, a finding that may be relevant to understanding the diverse functional effects of antipsychotic drugs.

Mack et al. reported a case of a young woman presenting with a 25 years history of dominant psychiatric symptoms, ranging from depression to typical schizophrenia with fluctuating psychotic features. The psychiatric symptoms were poorly responsive to various traditional psychotropic interventions and required multiple prolonged psychiatric hospitalizations. Throughout the course of the illness, she had intermittently exhibited mild skin rashes thought to be related to possible underlying mixed connective tissue disorder. Indeed, one of her later relapses was associated with severe generalize exanthema that resolved rapidly with steroid and azathioprine. KB (the editor above) suspected that the psychosis is likely related to an underling systemic immune disorder for which treatment with azathioprine was maintained, resulting in a complete remission of the psychiatric syndrome for approximately 16 years. However, upon azathioprine discontinuation due to pregnancy, the patient developed a severe relapse of psychosis accompanied by severe diffuse skin rashes that required a 2 years inpatient psychiatric care. Treatment with various immunosuppressants, including belimumab, resulted in a full remission of both psychosis and skin rashes. The combination of systemic biomarkers for immune activation and mild CSF inflammatory abnormalities including the presence of OCBs led to the final diagnosis of atypical Lupus erythematosus with CNS involvement presenting with dominant psychiatric manifestations mimicking schizophrenia.

Endres et al. report a case of steroid-responsive chronic psychosis associated with autoimmune thyroiditis mimicking antipsychotics-resistant schizophrenia, in the context of several clinical red flags that collectively pointed to underlying organic etiopathogenesis. These were intermittent electroencephalogram (EEG) slowing, mild temporal atrophy, and elevated thyroid antibodies. This case illustrates the importance of considering autoimmune psychosis in the differential diagnosis of secondary (organic) psychosis and to complete the relevant diagnostic workup to early identify this subgroup of immune-responsive psychosis. Endres et al. report another case with surprisingly rapid improvement of chronic schizophrenic symptoms under newly introduced antiepileptics after having identified respective suggestive EEG signs.

Ong et al. presented a case of primary Sjögren‘s syndrome with dominant severe obsessive-compulsive together with depressive symptoms requiring psychiatric hospitalization. The diagnosis was eventually made based on the presence of serum biomarkers of Sjögren‘s syndrome coupled with CSF findings of mild inflammation. A few months of treatment with various immunosuppressants together with plasmapheresis resulted in a complete remission of all neuropsychiatric symptoms. The authors suggest that the clinical approach to the management of psychotropic–resistant obsessive-compulsive disorder should also include a careful search for biomarkers of inflammation and autoimmunity.

Greenberg reports a case of severe psychotropic-refractory pediatric neuropsychiatric syndrome mimicking bipolar disorder thought to be linked to previously unrecognized infection, likely Bartonellosis. The presentation and the clinical course did not meet “pediatric autoimmune neuropsychiatric disorders associated with streptococcal infections” (PANDAS) criteria. The patient exhibited a clinically meaningful response to antibiotics targeting Bartonella infection. This case illustrates that prior clinically unrecognized infections can serve as immunological triggers of secondary psychiatric illnesses.

Klein provided a thorough review of the clinical and experimental evidence supporting the “viral hypothesis of schizophrenia.” The author suggests that HSV-1 infection of specific limbic brain regions such as hippocampal dentate gyrus among other factors is potentially mechanistically linked to psychosis in a subset of individuals with schizophrenia. The mechanisms potentially linking viral infections to the neurobiology of schizophrenia were highlighted. The author suggests that silencing the viral elements such as HSV-1, via either administering antiviral treatment or suppressing environmental factors that can influence viral expression such as stress can thereby potentially be curative in a subgroup of individuals with schizophrenia.

In summary, the data from these reports strengthen the evidence linking immune dysfunction, autoimmunity, and neuroinflammation to various primary psychiatric illnesses including schizophrenia and affective spectrum disorders. Alternatively, they also show that brain autoimmune disorders such as autoimmune encephalitis and autoimmune encephalopathy can also present with diverse neuropsychiatric syndromes masquerading as severe mental disorders such as primary psychotic disorders unresponsive to traditional psychotropic and behavioral interventions. Thus, it is critical to obtain a detailed history, perform a thorough examination, recognize clinical features (red flags) suggestive of organic causes, and complete a relevant diagnostic workup to include screening for relevant neuronal antibodies in serum and CSF to exclude other organic causes, in order not to overlook immunological causes of new-onset secondary (organic) neuropsychiatric syndromes. Collectively, the above data also support the ME hypothesis of schizophrenia and severe mental illness (1, 2) and the concept of autoimmune psychosis (3) mechanistically linking the neurobiology of a subset of psychosis to underlying inflammatory and immunological changes in the brain. Additional research studies investigating the prevalence of autoimmune psychosis among individuals with new-onset psychosis are needed. Further, development of an expert consensus on the evidence-based clinical practice guidelines addressing the diagnostic challenges and therapeutic dilemmas of new-onset psychosis of suspected immune origin is warranted.

## Author Contributions

KB prepared the intitial version of the Editorial, which was corrected and supervised and completed by SN and DB.

### Conflict of Interest Statement

The authors declare that the research was conducted in the absence of any commercial or financial relationships that could be construed as a potential conflict of interest.

## References

[B1] Bechter K. Updating the mild encephalitis hypothesis of schizophrenia. Prog Neuropsychopharmacol Biol Psychiatry. (2013) 42:71–91 10.1016/j.pnpbp.2012.06.019 22765923

[B2] Bechter K Mild encephalitis underlying psychiatric disorders-a reconsideration and hypothesis exemplified on Borna disease. Neurol Psychiatry Brain Res. (2001) 9:55–70. Available online at: http://www.elsevier-data.de/journals/Bechter_K_2001_ME_Hypothesis_NPBR.pdf

[B3] Najjar S Steiner J Najjar A Bechter K. A clinical approach to new-onset psychosis associated with immune dysregulation: the concept of autoimmune psychosis. J Neuroinflammation. (2018) 15:40. 10.1186/s12974-018-10607-y 29433523 PMC5809809

